# Racial/ethnic differences in risk factors for non-cardia gastric cancer: an analysis of the Multiethnic Cohort (MEC) Study

**DOI:** 10.1007/s10552-024-01934-9

**Published:** 2024-11-07

**Authors:** Alexandra Adams, Atish Gandhi, Patricia Friedmann, Srawani Sarkar, Brijesh Rana, Meira Epplein, Lynne Wilkens, Brian Z. Huang, Haejin In

**Affiliations:** 1 Division of Surgical Oncology, Rutgers Cancer Institute, New Brunswick, NJ USA; 2 Albert Einstein College of Medicine, Bronx, NY USA; 3 Department of Population Health Sciences, Duke University, Durham, NC USA; 4 University of Hawaiʻi Cancer Center, University of Hawaiʻi at Mānoa, Honolulu, USA; 5 Department of Population and Public Health Sciences at the Keck School of Medicine of USC, Los Angeles, USA; 6 Department of Health, Behavior and Policy, Rutgers University, Piscataway, NJ USA; 7 Rutgers Cancer Institute of New Jersey, 195 Little Albany Street, New Brunswick, NJ 08903 USA

**Keywords:** Noncardia gastric cancer, Heatlhcare disparities, Screening and risk stratification

## Abstract

**Purpose:**

Gastric cancer (GC) incidence rates show notable differences by racial/ethnic groups in the US. We sought to determine whether stratification by race/ethnicity would reveal unique risk factors for development of non-cardia gastric cancer (NCGC) for US population.

**Methods:**

Analysis included 1,112 incident cases of NCGC and 190,883 controls from the Multiethnic Cohort Study, a prospective US cohort study that recruited individuals living in Hawaii and California, aged 45–75 years from 5 races/ethnicities. Descriptive analysis and Cox regression models examined the association of risk factors for GC and calculate hazard ratios for each race/ethnicity, adjusting for sociodemographic and dietary variables.

**Results:**

Increasing age and male sex were risk factors for NCGC for most race/ethnicities. Higher risk was associated with: GC family history for Latino and Japanese American individuals [HRs range from 1.75 to 1.98]; foreign-born for Japanese American individuals [HR: 1.52, 95% CI 1.11–2.09]; lower education for African American, Japanese American, and Native Hawaiian individuals [HRs range from 1.30 to 1.74]; daily alcohol consumption for African American individuals[HR: 1.56, 95% CI 1.04–2.35]; current smoking for Latino and Japanese American individuals [HRs range from 1.89 to 1.94]; sodium consumption in the highest quartile for White individuals [HR: 2.55, 95% CI 1.23–5.26] compared to the lowest quartile; fruit consumption in the 2nd, 3rd, and 4th highest quartile for Native Hawaiian individuals [HRs range from 2.19 to 2.60] compared to the lowest quartile; diabetes for African American individuals [HR: 1.79, 95% CI 1.21–2.64]; and gastric/duodenal ulcers for Native Hawaiian individuals [HR: 1.82, 95% CI 1.04–3.18].

**Conclusion:**

Analyses by racial/ethnic group revealed differing risk factors for NCGC. Increased knowledge of the varying pathways to GC can support personalized GC prevention strategies and risk stratification tools for early detection.

**Supplementary Information:**

The online version contains supplementary material available at 10.1007/s10552-024-01934-9.

## Introduction

In 2023, it is estimated that there will be 26,500 new gastric cancer (GC) cases and 11,130 GC-related deaths in the United States (US) [1]. Unfortunately, only 28% of cases are expected to be diagnosed in localized stages when patients are asymptomatic and most likely to benefit from curative resection [2, 3]. In fact, up to 37% of GC in the US are detected at Stage 4, where five-year survival rate is only 5.9% [2]. Unlike Korea and Japan, where there are robust guidelines for GC screening, the US lacks a national program for GC prevention and control, which limits early detection and treatment [4].

A major factor behind the lack of screening guidelines is the low incidence of GC in the US, making population-based screening impractical [5]. However, while less common in the general population, GC incidence is markedly elevated in certain subsets of the population, with roughly double the incidence rates in individuals of Asian (20.8), African American (18.4), and Latino (17.1) race/ethnicity compared to White individuals (10.7 per 100,000 persons) [6]. Several studies have been published on cost-effectiveness of GC screening with endoscopy (EGD) in certain minority populations. One cost-effectiveness model found EGD with biopsies at time of screening colonoscopy for colon cancer (50 years) was cost-effective for non-Hispanic African American, Hispanic, and Asian individuals, though not for non-Hispanic White individuals [7]. This suggests that a stratified approach to GC screening could be effective.

An understudied aspect of GC is how risk factors vary by race/ethnicity in the US. Racial/ethnic disparities in GC incidence suggest host and environmental exposures may vary in different racial/ethnic subgroups. Discerning differences in risk factors, which in turn can help us better understand reasons for differences in GC incidence by race/ethnicity, would improve our ability to efficiently implement preventative screening based on individuals’ underlying risk. A study to examine differing GC risk factors by race/ethnicity would increase our knowledge of the varying pathways to GC.

In this study, we aim to determine whether there are differences in GC risk factors by race/ethnicity. Examining the magnitude of risk associated with race/ethnicity for GC and determining the most predictive risk factors for each racial/ethnic group will offer the evidence needed to inform the future development of a stratified GC risk assessment tool for tailored prevention and early detection efforts for the US population.

## Methods

### Study population

The Multiethnic Cohort (MEC) study recruited residents of Hawaii and Los Angeles from 1993 to 1996. The study enrolled men and women aged 45–75 years from five main racial/ethnic groups: African American, Japanese American, Latino, Native Hawaiian, and White. The cohort of over 215,000 participants completed a comprehensive questionnaire at time of enrollment. The questionnaire encompassed demographic factors, diet, medications, occupation, physical activity, first degree family history of cancer, and vitamin supplement intake. Detailed information about the cohort has been described elsewhere [8]. After the baseline survey, follow-up surveys were sent every 5 years and participants were followed for development of incident cancer. Incident cases of cancer were identified through two state-wide SEER registries: The Hawaii Tumor Registry and the California State Cancer Registry. For the purposes of this study, participants who were diagnosed with GC up until 2014 were considered cases, while those who were not were considered controls. Given the differences in pathogenesis of cardia (CGC) versus NCGC, the subtypes were divided and analyzed separately. However, due to the low number of CGC cases captured in this data set, only analysis of NCGC is reported. NCGC was defined using the ICD-O-3 site codes C16.1–9. Analysis excluding C16.8–9 (overlapping and unspecified) in the definition of NCGC was also examined.

### Data collection

Survey data obtained at the time of enrollment into MEC was used. Race and ethnicity were examined as 5 distinct population groups—White, African American, Latino, Japanese American, and Native Hawaiian. Variables included in the present analysis were first degree family history of GC (defined as mother, father, or full sibling having been diagnosed with GC), as well as personal demographics such as education (≤ 12 years or > 12 years), body mass index (BMI, ≤ 25, 25 to < 30, ≥ 30 kg/m^2^), smoking status (never smoker, former smoker, current smoker), and history of diabetes mellitus, intestinal polyps, and stomach or duodenal ulcers. Immigration status was categorized as foreign born or US born. Dietary variables included fruit and vegetable consumption (by quartile, gram/day) and sodium intake (by quartile, gram/day). Quartiles were based on distribution across the entire cohort. For alcohol consumption the data was analyzed as < 1 drink/day or ≥ 1 drink/day (1 drink = 14 g alcohol/day).

### Statistical analysis

MEC provided 201,064 records to the study team. After removing records with missing information about GC status (i.e., lost to follow-up), whether they were born in the US and time to event data, records from 192,186 individuals who participated in the study were included in the present analysis.

Categorical variables were examined by frequency and percentage, while median and interquartile range (IQR) were used for continuous variables. Tests for heterogeneity using Chi-square test were performed to assess differences for each variable across racial/ethnic groups.

The Age-adjusted incidence rates for non-cardia gastric cancer (NCGC) were calculated using direct method with the Year 2000 US standard population as the reference. Age-specific rates were computed by dividing the number of NCGC cases by the MEC total person-years for each age and race/ethnicity group. These rates were then multiplied by the corresponding standard population weight (proportion of the US population in that age group). The weighted rates were summed across all age groups to obtain the overall annual rates [9].

Cox regression models were created to estimate the hazard ratio (HR) and 95% confidence interval (CI) of developing NCGC from time of study enrollment until time of NCGC diagnosis or last follow-up for each racial/ethnic group and cancer subtypes. Reference groups for each variable are stratified by race/ethnicity and indicated in the tables where appropriate. Briefly, age was calculated as risk increase per year, overweight (> 25– < 30) and obese (≥ 30) BMIs were compared to the reference group of BMI ≤ 25 kg/m^2^, sodium and fruit consumption quartiles were compared to the 1st quartile, and foreign born were compared to US born using the defined categories above. Individuals with missing data were excluded from the final analysis. Any cell size less than 10 we have denoted as < 12% to preserve confidentiality and have removed corresponding variables from the models. All analyses were conducted by SAS 9.4 (SAS Institute, Inc., Cary, NC, USA).

## Results

Of the 192,186 records analyzed for this study, total number of GC was 1,303, of which 1,112 incident NCGCs were identified over a median follow-up of 20.3 years. Median follow-up time stratified by racial/ethnic group were similar for each group and can be found in Supplemental Table 1 and Table 1.

**Table 1:** Demographics, lifestyle and dietary information of participants with non-cardia GC and no GC by race/ethnicity

Variables	Categories	White Median follow-up 20.30 years		African American Median follow-up 19.75 years		Latino Median follow-up 20.70 years		Japanese American median follow-up 20.28 years		Native Hawaiian Median follow-up 20.24 years	
		No GC	NCGC	No GC	NCGC	No GC	NCGC	No GC	NCGC	No GC	NCGC
		*n* = *47,167*	*n* = *80*	*n* = *32,869*	*n* = *166*	*n* = *43,262*	*n* = *249*	*n* = *53,871*	*n* = *527*	*n* = *13,714*	*n* = *80*
Age	Median (IQR)	59 (51–67)	**68** (63–72)*	62 (54–69)	**66** (60–71)*	60 (54–65)	**63** (58–68)*	62 (53–69)	**68** (63–72)*	55 (49–63)	**61.5** (55–67)*
Sex	Male	46.2%	53.8%	36.5%	50.6%*	48.1%	55.8%*	47.0%	60.1%*	43.4%	56.3%*
Foreign (non-US) born	Yes	11.6%	< 12%**	3.7%	< 12%**	49.9%	50.6%	8.4%	8.5%	0.4%	< 12%**
Family history of GC	Yes	2.9%	< 12%**	2.0%	< 12%**	3.7%	6.4%*	9.4%	19.3%*	4.5%	< 12%**
Education	≤ 12grade	6.6%	19.0%*	14.6%	20.6%*	45.8%	50.8%	9.3%	15.7%*	12.4%	15.2%
	missing	1.2%	0.7%	1.5%	0.6%	1.8%	1.2%	0.74%	**0.7%**	0.9%	1.2%
BMI	< 25	46.0%	41.0%	26.9%	26.7%	28.2%	31.2%	60.2%	65.1%	26.6%	32.9%
	25- < 30	36.5%	42.3%	41.0%	48.5%	46.7%	47.8%	32.8%	30.0%	37.5%	39.2%
	> 30	17.6%	16.7%	32.1%	24.8%	25.1%	21.1%	7.0%	4.9%	35.9%	27.9%
	missing	0.28%	2.50%	3.20%	3.01%	0.54%	0.38%	0.54%	0.38%	0.88%	1.25%
Alcohol consumption	≥ 1 drink/d	27.7%	21.2%	13.0%	20.5%*	13.4%	11.6%	12.4%	16.9%*	16.2%	26.2%*
Smoking	Never	38.8%	40.5%	37.6%	32.7%	48.7%	41.9%*	50.3%	40.1%*	39.1%	28.8%
	Former	44.7%	46.8%	40.2%	40.6%	37.3%	37.8%*	38.0%	45.6%*	38.5%	48.8%
	Current	16.5%	12.7%	22.3%	26.7%	14.0%	20.3%*	11.7%	14.3%*	22.4%	22.5%
	missing	0.8%	1.2%	1.5%	0.6%	3.3%	3.2%	0.9%	0.2%	0.9%	0.0%
Sodium consumption	Q1	26.4%	18.8%	37.4%	35.5%	22.2%	21.3%	20.5%	21.4%	17.5%	< 12%**
	Q2	28.0%	30.0%	23.9%	20.5%	21.4%	21.3%	27.3%	25.7%	20.3%	22.5%*
	Q3	26.2%	25.0%	19.8%	23.5%	23.0%	24.1%	28.6%	29.0%	25.0%	< 12%***
	Q4	19.4%	26.2%	18.9%	20.5%	33.4%	33.3%	23.6%	24.9%	37.3%	51.3%*
Fruit consumption	Q1	24.3%	27.5%*	26.9%	25.9%	22.9%	20.9%	25.4%	21.1%*	27.9%	13.8%*
	Q2	27.0%	16.3%*	24.2%	27.1%	23.2%	25.7%	25.6%	24.7%*	23.6%	22.5%*
	Q3	27.0%	20.0%*	23.6%	20.5%	23.3%	21.7%	26.3%	25.8%*	22.0%	23.8%*
	Q4	21.7%	26.2%*	25.4%	26.5%	30.6%	31.7%	22.7%	28.5%*	26.4%	40.0%*
DM	Yes	5.9%	12.5%*	15.8%	21.1%	15.7%	16.5%	10.5%	11.9%	15.0%	12.5%
Presence of ulcers	Yes	11.0%	15.0%	12.8%	14.5%	12.1%	14.1%	12.2%	17.3%*	10.5%	21.2%*
Presence of polyps	Yes	6.5%	15.0%*	4.5%	7.2%	3.5%	4.8%	8.4%	9.1%	4.2%	< 12%**

*NCGC* non-cardia gastric cancer, *IQR* interquartile range, *GC* gastric cancer, *BMI* body mass index, *drink/d* drink per day, *Q1* first quartile, *Q2* second quartile, *Q3* third quartile, *Q4* fourth quartile, *DM* diabetes

*Indicates *p* < 0.05 compared to No GC

**Cells containing less than 10 patients are demoted as < 12% for confidentiality

***Additional cell suppressed for confidentiality

Bolded values with * indicates p<0.05

### Differences in anatomic distribution of GC and difference in risk factors by race/ethnicity

**Fig. 1:**
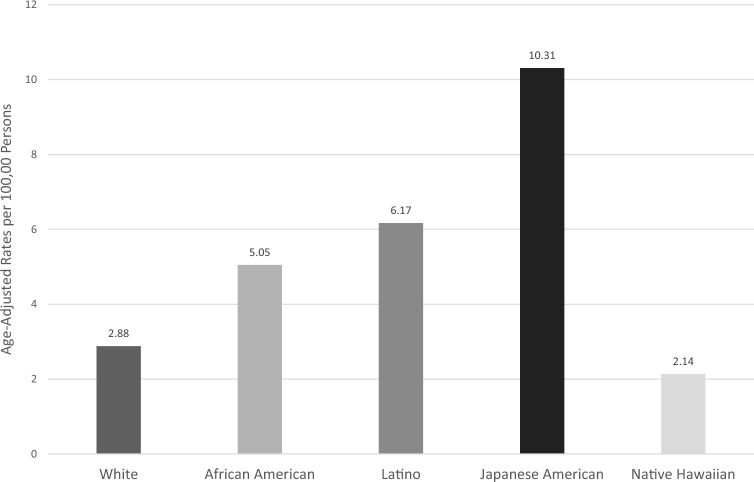
Age adjusted rates (AAR) for GC by race/ethnicity during study period (1993–2014)

The rates of NCGC varied widely by racial/ethnic group. The age-adjusted incidence rates for NCGC was lowest for Native Hawaiian individuals at 2.13 per 100,000 followed by White individuals at 2.88 per 100,000. All other groups had markedly higher rates, with Japanese American individuals having the highest rate at 10.31 per 100,000, and rate for African American (5.05) and Latino (6.17) individuals falling between rates in White and Japanese American individuals (Fig. 1). By subtype NCGC was predominant for all racial/ethnic groups, accounting for 85–90% of all GC for all racial/ethnic groups except White individuals, for which NCGC comprised 67% of all GCs.

Tests for heterogeneity showed differences were present by race/ethnic groups for all variables examined (Supplemental Table 1).

### Descriptive comparisons and multivariable models

Descriptive analyses of risk factors for NCGC by race/ethnicity are shown in Table 1. Compared to White individuals who did not develop GC, White individuals who developed NCGC tended to be older, have or have less than a 12th grade education, diabetes, and report history of having intestinal polyps. While differences were noted in consumption of fruit, NCGC was more common in the first and fourth quartile, and risk was not linear. African American individuals who developed NCGC were older, more likely to be male, have or have less than a 12th grade education, and report having one or more drinks per day; Latino individuals who developed NCGC were older, more likely to be male, have a first degree family history of GC, and be current smokers than Latino individuals who didn’t develop GC; Japanese American individuals who developed NCGC were older, more likely to be male, have a first degree family history of GC, have or have less than a 12th grade education, report having one or more drinks per day, be current or former smokers, report having higher fruit consumption, and report history of ulcers; and Native Hawaiian NCGC cases were older, more likely male, report having one or more drinks per day, report having higher fruit consumption, and report history of ulcers.

Table 2 shows results of the multivariable cox regression models for the risk of NCGC for each racial/ethnic group. These models together show that increasing age was a common risk factor for NCGC for all race/ethnicities, as was male sex, with the exception of White individuals. Higher risk for NCGC was uniquely associated with GC first degree family history for Latino [HR: 1.75, 95% CI 1.05–2.91] and Japanese American individuals [HR: 1.98, 95% CI 1.60–2.47]; lower education for African American [HR: 1.65, 95% CI 1.20–2.27], Japanese American [HR: 1.30, 95% CI 1.08–1.56], and Native Hawaiian individuals [HR: 1.74, 95% CI 1.06–2.87]; daily alcohol consumption for African American individuals [HR:1.57, 95% CI 1.05–2.36]; current smoking for Latino [HR: 1.94, 95% CI 1.34–2.79] and Japanese American individuals [HR: 1.89, 95% CI 1.42–2.52] compared to never smokers; and highest sodium consumption quartile for White [HR: 2.55, 95% CI 1.23–5.26] individuals compared to the lowest. Being foreign-born was a risk factor only for Japanese American individuals [HR: 1.52, 95% CI 1.11–2.09], having diabetes only for African American individuals [HR: 1.79, 95% CI 1.21–2.64], and having ulcers only for Native Hawaiian individuals [HR 1.82, 95% CI 1.04–3.18]. Increased fruit consumption was associated with a decreased risk in White individuals [Q2 HR 0.44 (95% CI 0.22–0.89; Q3 HR 0.43 (95% CI 0.22–0.85) compared to Q1], but was associated with a higher risk of GC in all quartiles for Native Hawaiian individuals [Q2 HR: 2.19, 95% CI 1.01–4.77; Q3 HR: 2.49, 95% CI 1.15–5.41; Q4 HR: 2.60, 95% CI 1.21–5.53 compared to Q1].

**Table 2:** Non-cardia GC prediction models, for each race/ethnicity

Risk factors	Categories	White *n* = *46,694* (80 cases) HR (CI)	African American *n* = *31,458* (166 cases) HR (CI)	Latino *n* = *41,385* (249 cases) HR (CI)	Japanese American *n* = *53,590* (527 cases) HR (CI)	Native Hawaiian *n* = *13,527* (80 cases) HR (CI)
Age	Risk per year increase	1.14(1.10–1.17)*	1.07(1.04–1.09)*	1.07(1.05–1.09)*	1.09(1.08–1.11)*	1.06(1.03–1.09)*
Sex	Male	1.27(0.78–2.06)	1.67(1.19–2.34)*	1.33(1.00–1.78)*	1.70(1.37–2.09)*	1.79(1.11–2.90)*
Foreign(non-US) born	Yes	N/A**	N/A**	1.09(0.84–1.42)	1.52(1.11–2.09)*	N/A**
Family history of GC	Yes	N/A**	N/A**	1.75(1.05–2.91)*	1.98(1.60–2.47)*	N/A**
Education	≤ 12 grade	1.40(0.87–2.23)	1.65(1.20–2.27)*	0.98(0.73–1.30)	1.30(1.08–1.56)*	1.74(1.06–2.87)*
BMI	25- < 30 (ref ≤ 25)	1.06(0.64–1.74)	1.12(0.77–1.64)	0.90(0.67–1.21)	0.90(0.73–1.08)	0.76(0.45–1.30)
	≥ 30 (ref ≤ 25)	0.97(0.50–1.89)	0.87(0.55–1.36)	0.85(0.58–1.23)	1.00(0.67–1.50)	0.75(0.42–1.36)
Alcohol consumption	≥ 1 drink/d	0.70(0.40–1.22)	1.57(1.05–2.36)*	0.76(0.51–1.14)	1.22(0.95–1.56)	1.59(0.93–2.71)
Smoking	Former (ref = never)	1.05(0.64–1.73)	0.99(0.68–1.45)	1.11(0.82–1.50)	1.34(1.09–1.66)*	1.46(0.86–2.50)
	Current (ref = never)	1.15(0.55–2.41)	1.43(0.93–2.21)	1.94(1.34–2.79)*	1.89(1.42–2.52)*	1.69(0.88–3.24)
Sodium consumption	Q2 (ref = Q1)	1.78(0.91–3.48)	0.91(0.59–1.41)	1.05(0.71–1.56)	0.82(0.63–1.05)	N/A**
	Q3 (ref = Q1)	1.78(0.88–3.59)	1.25(0.82–1.91)	1.07(0.73–1.58)	0.86(0.67–1.11)	N/A**
	Q4 (ref = Q1)	2.55(1.23–5.26)*	1.07(0.67–1.72)	1.02(0.70–1.50)	0.85(0.65–1.11)	N/A**
Fruit consumption	Q2 (ref = Q1)	0.44(0.22–0.89)*	1.07(0.70–1.64)	1.15(0.79–1.67)	1.02(0.78–1.32)	2.19(1.01–4.77)*
	Q3 (ref = Q1)	0.43(0.22–0.85)*	0.82(0.52–1.32)	0.89(0.59–1.34)	0.97(0.75–1.27)	2.49(1.15–5.41)*
	Q4 (ref = Q1)	0.89(0.49–1.61)	1.01(0.64–1.59)	1.06(0.77–1.57)	0.85(0.65–1.11)	2.60(1.21–5.53)*
DM	Yes	1.90(0.93–3.88)	1.79(1.21–2.64)*	1.10(0.77–1.57)	1.11(0.85–1.46)	0.90(0.46–1.77)
Presence of Ulcers	Yes	1.07 (0.56–2.04)	1.06(0.68–1.66)	1.03(0.70–1.50)	1.18(0.94–1.48)	1.82(1.04–3.18)*
Presence of Polyps	Yes	1.81(0.97–3.39)	1.41(0.76–2.62)	1.16(0.63–2.14)	0.80(0.59–1.08)	N/A**

*HR* hazard ratio, *CI 95%* confidence interval, *GC* gastric cancer, *BMI* body mass index, *drink/d* drink per day, *Q1* first quartile, *Q2* second quartile, *Q3* third quartile, *Q4* fourth quartile, *DM* diabetes

Age, Sex, Foreign (non-US) born, Family history of GC, Education, BMI, Alcohol Consumption, Smoking, Sodium and Fruit consumption, DM, Presence of Ulcers, Presence of Polyps were the variables included in the backward selection model

*Indicates *p* < 0.05 compared to No GC

**Variables with cells containing less than 10 patients were removed from the final model

Alternative definition of NCGC excluding C16.8–9 (overlapping and unspecified) resulted in exclusion of 24.7% patients from the original definition (NCGC total *n* = 1102 vs *n* = 840). The multivariable model with this subset of individuals showed minor differences in results compared to the broader definition. Male sex remained significant for African American, Native Hawaiian, and Japanese American individuals but was no longer statistically significant for Latino individuals. Family history remained significant for Japanese American individuals, but no longer statistically different for Latino individuals. Education remained significant for African Americans and Native Hawaiian individuals but was no longer significant for Japanese American individuals. Alcohol consumption was no longer significant for individuals of any race/ethnicity. Highest quartile sodium consumption was no longer significant for White individuals compared to first quartile. The presence of ulcers was no longer significant for any individuals of any race/ethnicity (Supplemental Table 2).

## Discussion

Using MEC, one of the largest prospective databases that was designed to enroll and study risk factors of individuals from racial/ethnic groups, our study shows that while some risk factors are common, many risk factors are unique among individuals from different racial/ethnic groups. While difference in incidence rates for NCGC among different races and ethnicities is well established, studies that explore differences in specific risk factors by racial/ethnic group are lacking [6, 7].

Consistent with published data, we found NCGC to be the dominant subtype for all racial/ethnic groups (67% for white individuals, 85–90% for all other groups) [10]. Given the distinct etiologies of these two GC subtypes, and the low number of CGC in this cohort, we felt it prudent to focus our analysis on NCGC. With few exceptions, increasing age and male sex were uniformly significant risk factors across all racial/ethnic groups, a phenomenon that has been well studied in GC [11–14]. Interestingly, however, there were also risk factors unique to individuals from each racial/ethnic group. High sodium consumption is a known risk factor for NCGC as it promotes colonization of *H. Pylori (HP)* as well as irritates the stomach wall, promoting gastric carcinogenesis [15–17]. Three recent meta-analyses all found an increased risk of NCGC associated with increased sodium or salt intake, ranging from 1.68 to 2.24 [18–20]. Yet, in our study, high sodium intake was found to be a risk factor for NCGC only for White individuals while it was not significantly associated with risk for African American, Latino, Native Hawaiian, and Japanese American individuals. This could be due to the fact that this was a US study, and thus, the breadth of foods included in the food frequency survey might not adequately cover the diverse range of diets in this country. For example, Japanese eat a wide variety of pickled foods that may not have been adequately captured in the MEC food frequency questionnaire but were included in Japanese-based studies where these diets are common. Conversely, bread, a high-salt item, was included in this survey and might have better captured salt intake for racial/ethnic groups in the US that favor bread as the predominant source of carbohydrate consumption. Furthermore, despite most studies utilizing food frequency questionnaires to determine sodium consumption, the definition of “high salt consumption” is not standardized across all studies, and perhaps the definition of “high salt consumption” used in this analysis is more stringent than other published studies. A limitation specific to the MEC questionnaire was that participants were asked to recall their average consumption of salt over the past year, which could be difficult to estimate, resulting in measurement error, whereas questionnaires included in the other cited studies only specified a recall time of 1 week.

Cigarette smoke is known to contain N-nitroso-compounds, thought to be involved in gastric carcinogenesis [21]. Several studies have looked at the association of smoking with GC, mainly focusing on the difference in risk between men and women. One analysis using MEC data found the association between smoking and CGC was significant for both men (HR 3.97) as well as women (HR 3.58), among all racial/ethnic groups combined [22]. Interestingly, a systematic review of 42 cohort studies cited increased risk of GC in former smokers relative to never smokers only in males (RR 1.34, 95% CI 1.22–1.47), but not females (RR 1.16, 95% CI 0.92–1.46) [23]. The same study found current smoking was associated with increased risk of CGC (RR 1.87; 95% CI 1.31–2.67) as well as NCGC (RR 1.60; 95% CI 1.41–1.80), albeit to a lesser extent. Similarly, our study did find smoking to be associated with NCGC for African American, Latino, and Japanese American individuals.

In the present study, diabetes was a strong risk factor only for African American individuals. This subset of the population also had the highest rates of diabetes overall among all racial/ethnic groups. The link between diabetes mellitus type II (DMII) and GC has been suggested in other population-based studies, though these studies suggest the effect is most significant in Asian populations and among women [24]. One hypothesis is that this link is due to shared risk factors between GC and DM, such as obesity, poor health literacy, lower socioeconomic status, and smoking status. However, our data differ from the recent meta-analysis of 14 studies by Dabo et al., which found no association between DMII and GC, even when controlling for *HP* seropositivity, age, sex, BMI, smoking status, alcohol consumption, fruit/vegetable intake, gastric cancer histologic type, and source of controls [25]. It is important to note that only two of the studies in this meta-analysis were from the US and the authors did not stratify patients by racial/ethnic groups.

For Latino individuals, as well as Japanese American individuals, GC family history was a significant risk factor. Several studies have documented increased risk of GC in those with a family history, especially in first degree relatives [26–28]. One reason for this can be attributed to a genomic syndrome, such as hereditary diffuse gastric cancer; however, this only accounts for about 1–3% of GC cases [29]. Another reason that GC is associated with family history is that gastric cancer is largely linked to infection with *HP*, which has been found to be transmissible from mother to child and is commonly found in families [30–32]. A recent study found that when patients with a family history of GC were treated for *HP*, their risk of GC was greatly reduced, supporting the idea that GC family history increases GC risk through greater likelihood of *HP* infection [33]. In an estimated 75% of NCGC cases, *HP* is the inciting event that triggers the Correa pathway, causing inflammation of the stomach leading to chronic gastritis, atrophic gastritis, intestinal metaplasia, dysplasia, and finally, cancer [34]. However, it is important to note that knowledge of family history of cancer can vary by population, which might explain why we did not find a significant association among the other racial/ethnic groups.

Although this study utilized one of the largest and most diverse prospective studies of lifestyle and dietary risk factors and subsequent development of cancer, there were several limitations in this study. A significant limitation was that the participants were from two primary locations: Los Angeles, California and Hawaii. The origins and ethnicities that represent each of the racial/ethnic combinations reflect those that reside in these study locations. For example, the majority of the Hispanic population in LA identify as Mexican, whereas the majority of the Hispanic population in the Northeast are individuals who identify as Puerto Rican and the majority of the population residing in Miami, FL are individuals who identify as Cuban [35]. Similarly, although this study enrolled Japanese Americans, the make-up of the Asian population across the country differs by region as well [36]. Furthermore, in contrast to SEER data, the incidence rates for NCGC in the MEC cohort were lower. As SEER does not specifically report disaggregated rates in Japanese Americans or Native Hawaiians, we are limited to comparisons made to race/ethnicity specific rates in White, African American, and Latino individuals. Rates in the MEC cohort for NCGC were roughly half the rates reported by SEER for White (2.8 vs 5.7), African American (5.1 vs 10.2), and Latino (6.2 vs 10.5) individuals [6]. This is likely due to the MEC cohort being select participants who have agreed to participate in a research study and are likely to have healthier lifestyle and dietary habits than the general population. Nonetheless, the magnitude of the difference in incidence rate between white and other races remain the same.

Generalization of these results to other regions of the country should be interpreted with caution. It is important to note that the categorization of race as presented here includes a heterogenous group of people from many different backgrounds. For example, “White” individuals can include people from Eastern Europe, where there are higher rates of GC, as well as people from Western Europe where there are typically lower rates of GC. “African American” individuals could potentially originate from not only Africa, but the Caribbean as well, which similarly have disparate rates of GC (lower in Africa, higher in the Caribbean). Finally, “Native Hawaiian and Pacific Islander” individuals include not only Polynesians, but also Micronesian and Melanesian backgrounds and people originating from Guam or Samoa [37]. In addition, racial/ethnic categories were based on self-reported information, which could have led to categorization error. Intestinal polyps were included in the analysis because gastric polyps are a risk factor for GC. However, this variable is likely capturing history of colonic polyps as it is more common than gastric polyps. As such, our finding that there was no increased risk with intestinal polyps likely reflects lack of association with colonic polyps, not gastric polyps. Finally, the MEC cohort were not surveyed on *HP* status, the largest risk factor for NCGC.

Latino and Asian American individuals represent the largest and fastest-growing racial/ethnic population in the US [35, 36]. This growing population, coupled with the known high incidence of gastric cancer in African American, Latino, and Asian-Pacific Islander populations means GC will continue to be one of the leading contributors to cancer health disparities in the US. While risk prediction models have been created in other countries, such as Japan and China, these populations are more homogenous than our US population [38–40]. In previous work, we showed that including race increases the predictive ability of a risk stratification model for a US population. Specifically, we found the addition of US generation, race, and cultural food consumption at ages 15–18 years improved the AUC for GC of the Harvard Cancer Index from 0.87 to 0.91 [41]. The US population as a whole is heterogenous, and there is need to understand differences in GC risk by race and develop risk models that consider the unique heterogeneity within each racial/ethnic group, and the varying associations with risk.

## Conclusion

This study improves our understanding of NCGC risk factors both common and unique to racial/ethnic groups in Southern California and Hawaii. While some risk factors were common, there were other risk factors unique to certain race/ethnicities. Increased knowledge of the varying pathways to NCGC can support personalized GC prevention strategies and GC risk stratification tools for early detection.

## Supplementary Information

Below is the link to the electronic supplementary material.

Supplementary file1 (DOCX 20 kb)

## Data Availability

For information on how to gain access to the multiethnic cohort, please see: https://www.uhcancercenter.org/for-researchers/mec-data-sharing.
