# Supplementary material for: Racial/ethnic differences in risk factors for non-cardia gastric cancer: an analysis of the Multiethnic Cohort (MEC) Study
Source: Cancer Causes Control. 2024 Nov 7;36(3):255–63. doi: 10.1007/s10552-024-01934-9 (PMC11928376; doi:10.1007/s10552-024-01934-9)
Supplement: Supplementary file 1 — Supplementary file1 (DOCX 20 kb) [file 10552_2024_1934_MOESM1_ESM.docx]

**Supplement table 1: Test for Heterogeneity**

|  |  | **White**  **N = 47287** | **African American**  **N = 33058** | **Latino**  **N = 43557** | **Japanese American**  **N = 54481** | **Native Hawaiian**  **N = 13803** | **P-Value*** |
| --- | --- | --- | --- | --- | --- | --- | --- |
| **% developed cancer** | Yes | 0.28% | 0.57% | 0.67% | 1.11% | 0.64% |  |
| **Median Follow-up** | Years | 20.30 | 19.75 | 20.70 | 20.28 | 20.22 |  |
| **Age** | Median (IQR) | 59  (51-67) | 63  (54-69) | 60  (54-66) | 62  (53-69) | 55  (49-63) | <0.0001 |
| **Sex** | Male | 46.25% | 36.61% | 48.20% | 47.20% | 43.52% | <0.0001 |
| **Foreign (non-US) Born** | Yes | 11.62% | 3.70% | 49.90% | 8.45% | 0.46% | <0.0001 |
| **Family History of GC** | Yes | 2.95% | 2.02% | 43.77% | 9.55% | 4.46% | <0.0001 |
| **Education** | ≤12grade | 73.54% | 58.62% | 31.17% | 59.82% | 46.82% | <0.0001 |
| **BMI** | <25 | 45.96% | 26.86% | 28.19% | 60.27% | 26.58% | <0.0001 |
|  | 25-<30 | 36.46% | 41.05% | 46.76% | 32.75% | 37.55% |  |
|  | >30 | 17.58% | 32.09% | 25.05% | 6.98% | 35.88% |  |
| **Alcohol consumption** | ≥1 drink/d | 27.67% | 13.02% | 13.40% | 12.50% | 16.24% | <0.0001 |
| **Smoking** | Never | 38.8% | 37.51% | 48.57% | 50.12% | 39.00% | <0.0001 |
|  | Former | 44.71% | 40.20% | 37.34% | 38.13% | 38.60% |  |
|  | Current | 16.50% | 22.30% | 14.10% | 11.75% | 22.40% |  |
| **Sodium Consumption** | Q1 | 26.36% | 37.42% | 22.23% | 20.50% | 17.40% | <0.0001 |
|  | Q2 | 28.02% | 23.86% | 21.34% | 27.26% | 20.28% |  |
|  | Q3 | 26.20% | 19.8% | 23.03% | 28.60% | 25.00% |  |
|  | Q4 | 19.42% | 18.92% | 33.40% | 23.66% | 37.40% |  |
| **Fruit Consumption** | Q1 | 24.31% | 26.86% | 22.89% | 25.41% | 27.85% | <0.0001 |
|  | Q2 | 27.00% | 24.20% | 23.22% | 25.60% | 23.60% |  |
|  | Q3 | 27.00% | 23.55% | 23.30% | 26.30% | 22.05% |  |
|  | Q4 | 21.77% | 25.40% | 30.60% | 22.70% | 26.51% |  |
| **DM** | Yes | 5.91% | 15.87% | 15.74% | 10.55% | 14.98% | <0.0001 |
| **Presence of Ulcers** | Yes | 10.97% | 12.82% | 12.15% | 12.30% | 10.54% | <0.0001 |
| **Presence of Polyps** | Yes | 6.55% | 4.46% | 3.50% | 8.43% | 4.25% | <0.0001 |

*Chi-square test P-value for heterogeneity

**Supplement table 2: Sensitivity Analysis – Alternative Definition of Non-cardia, excluding ICD0-3 site codes C168 (overlapping sites, n=125) and C169 (NOS, n=137)**

| Risk Factors | Categories | White  *n=46694*  (80 cases)  HR (CI) | African American  *n=31458*  (166 cases)  HR (CI) | Latino  *n=41385*  (249 cases)  HR (CI) | Japanese American  *n=53590*  (527 cases)  HR (CI) | Native Hawaiian  *n=13527*  (80 cases)  HR (CI) |
| --- | --- | --- | --- | --- | --- | --- |
| Age | Risk per year increase | 1.15(1.10-1.20)* | 1.07(1.04-1.09)* | 1.07(1.48-1.09)* | 1.10(1.09-1.12)* | 1.06(1.03-1.10)* |
| Sex | Male | 1.16(0.64-2.08) | 1.61(1.09-2.37)* | 1.29(0.94-1.78) | 1.72(1.34-2.19)* | 1.87(1.06-3.29)* |
| Foreign(non-US) born | Yes | N/A** | N/A** | 1.16(0.86-1.56) | 1.72 (1.21-2.43)* | N/A** |
| Family History of GC | Yes | N/A** | N/A** | 1.66(0.92-2.99) | 2.10(1.65-2.65)* | N/A** |
| Education | ≤12 grade | 1.56(0.89-2.75) | 1.66(1.15-2.40)* | 1.02(0.74-1.42) | 1.21(0.93-1.50) | 1.98(1.09-3.61)* |
| BMI | 25-<30 (ref ≤25) | 0.97(0.53-1.77) | 1.37(0.87-2.16) | 0.87(0.62-1.21) | 0.89(0.71-1.11) | 0.69(0.37-1.28) |
|  | ≥30 (ref ≤25) | 0.81(0.35-1.87) | 1.13(0.67-1.91) | 0.86(0.57-1.29) | 0.92(0.57-1.50) | 0.71(0.35-1.41) |
| Alcohol consumption | ≥1 drink/d | 0.58(0.29-1.19) | 1.52(0.94-2.44) | 0.75(0.47-1.19) | 1.24(0.94-1.63) | 1.54(0.82-2.89) |
| Smoking | Former (ref=never) | 1.29(0.70-2.40) | 1.11(0.72-1.72) | 1.17(0.83-1.65) | 1.41(1.11-1.80)* | 1.46(0.78-2.72) |
|  | Current (ref=never) | 1.57(0.66-3.74) | 1.61(0.98-2.66) | 2.06(1.37-3.10)* | 2.06(1.49-2.85)* | 1.68(0.79-3.60) |
| Sodium consumption | Q2 (ref=Q1) | 1.32(0.61-2.89) | 0.87(0.52-1.45) | 1.02(0.66-1.58) | 0.90(0.66-1.21) | N/A** |
|  | Q3 (ref=Q1) | 1.56(0.70-3.45) | 1.48(0.92-2.37) | 1.08(0.70-1.66) | 0.95(0.71-1.27) | N/A** |
|  | Q4 (ref=Q1) | 1.94(0.83-4.55) | 1.04(0.60-1.81) | 0.99(0.64-1.51) | 0.96(0.71-1.31) | N/A** |
| Fruit consumption | Q2 (ref=Q1) | 0.41(0.18-0.93)* | 1.17(0.71-1.93) | 1.13(0.75-1.71) | 1.05(0.78-1.41) | 2.66(1.01-7.03)* |
|  | Q3 (ref=Q1) | 0.35(0.15-0.80)* | 1.03(0.61-1.75) | 0.73(0.46-1.17) | 0.969(0.72-1.31) | 3.29(1.26-8.57)* |
|  | Q4 (ref=Q1) | 0.77(0.37-1.56) | 1.01(0.59-1.73) | 1.1(0.72-1.66) | 1.24(0.92-1.67) | 4.07(1.62-10.23)* |
| DM | yes | 2.25(0.99-5.12) | 1.66(1.06-2.60)* | 1.24(0.84-1.83) | 1.07(0.78-1.46) | 0.84(0.37-1.88) |
| Presence of Ulcers | yes | 0.95 (0.43-2.13) | 1.20(0.73-1.96) | 1.15(0.77-1.72) | 1.14(0.88-1.49) | 1.67(0.86-3.26) |
| Presence of Polyps | yes | 1.82(0.84-3.91) | 0.80(0.33-1.97) | 1.20(0.61-2.36) | 0.70(0.49-1.01) | N/A** |

*Indicates P<0.05 compared to No GC

** Variables with cells containing less than 10 patients were removed from the final model

HR hazard ratio; CI 95% confidence interval; GC gastric cancer; BMI Body Mass Index; drink/d drink per day; Q1 first quartile; Q2 second quartile; Q3 third quartile; Q4 fourth quartile; DM diabetes.

Age, Sex, Foreign (non-US) born, Family history of GC, Education, BMI, Alcohol Consumption, Smoking, Sodium and Fruit consumption, DM, Presence of Ulcers, Presence of Polyps were the variables included in the backward selection model.
